# Supplementary material for: Methylation and PTEN activation in dental pulp mesenchymal stem cells promotes osteogenesis and reduces oncogenesis
Source: Nat Commun. 2019 May 20;10:2226. doi: 10.1038/s41467-019-10197-x (PMC6527698; doi:10.1038/s41467-019-10197-x)
Supplement: Supplementary file 1 — Supplementary Information [file 41467_2019_10197_MOESM1_ESM.pdf]

Methylation and PTEN activation in dental pulp mesenchymal stem cells promotes osteogenesis and reduces oncogenesis

Shen et al.

Supplementary Information

Inventory:
Supplementary Figure 1 – Supplementary Figure 7.

Supplementary Table 1 – Supplementary Table 3

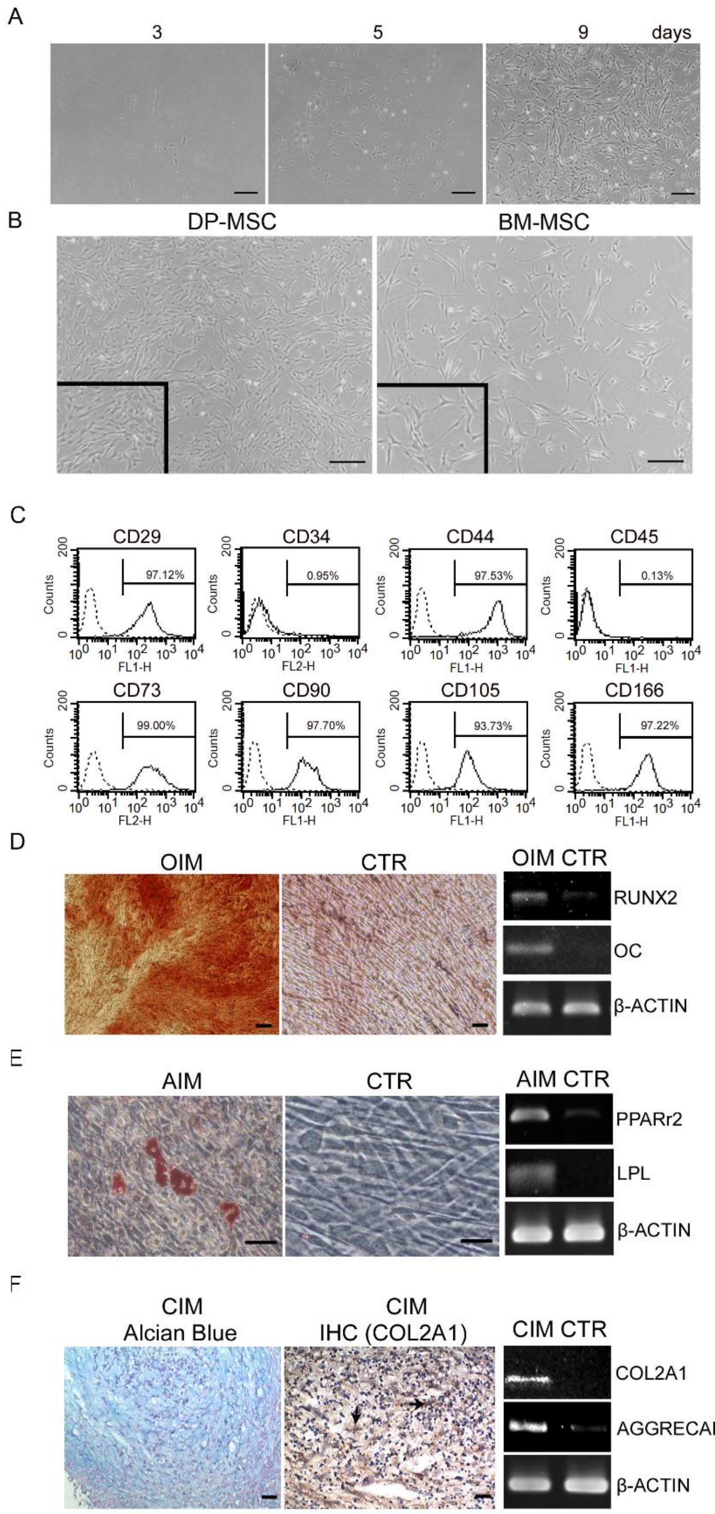

Supplementary Figure 1. Isolation and characterization of DP-MSCs. DP-MSCs were isolated form the pulp of normal exfoliated human deciduous incisors. (A) Morphology of DP-MSCs at indicated days of culture. (B) Both DP-MSCs and BM-MSCs adopt fibroblast-like morphology at same passage. The cell size is smaller in DP-MSCs than that in BM-MSCs. (C) Surface protein profiles of DP-MSCs as analyzed by flow cytometry. (D-F) The differentiation potency of

DP-MSCs. Cells induced in (D) osteogenic induction medium (OIM, 2 weeks), (E) adipogenic induction medium (AIM, 5 weeks) and (F) chondrogenic induction medium (CIM, 3 weeks), were analyzed with (D) Alizarin Red S, (E) Oil Red O, (F) Alcian Blue staining and immunohistochemistry of type II collagen (COL2A1), respectively. (D-F, Right) Quantitative RT-PCR for mRNA levels of differentiation makers. (D) RUNX2 and OC, (E) PPARγ2 and LPL, (F) AGGRECAN and COL2A1 were detected by RT-PCR in OIM, AIM and CIM, respectively. Bar = 250 μm.

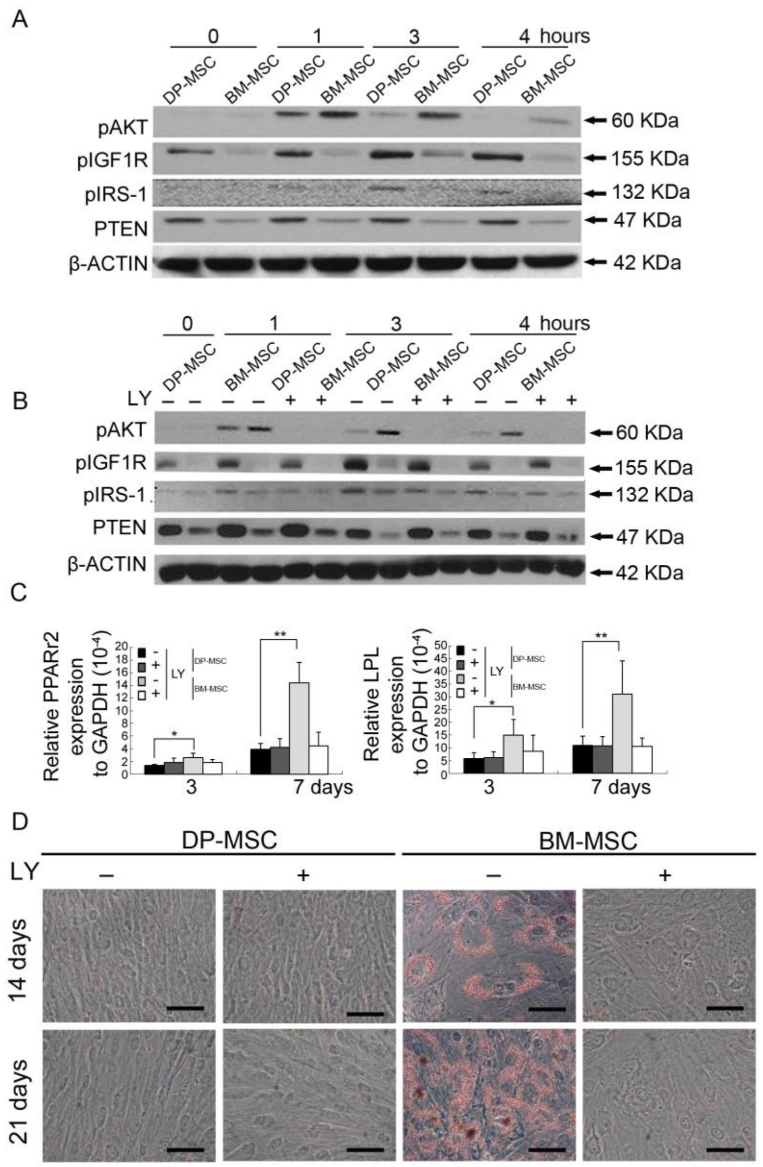

Supplementary Figure 2. DP-MSCs decrease in insulin signaling with high PTEN expression. DP-MSCs and BM-MSCs were cultured in serum-free basal medium for 1 day. (A) Cells were then treated with serum-free adipogenic induction medium for up to 4 h, and analyzed by western blotting. (B) Cells were first pretreated with or without PI3K inhibitor, LY294002 (LY, 50μM), for 30 minutes and then treated in serum-free adipogenic induction medium for up to 4 h, and analyzed by western blotting (upper). (C and D) Cells were pretreated with or without LY294002 (LY, 50μM), for 30 minutes and then treated in adipogenic induction medium for up to 3 and 7 days. Cells were analyzed by (C) quantitative RT-PCR, (D, Left) Oil Red O staining, and (D, Right) quantification of the Oil Red O extract by measuring the O.D. at 510 nm at indicated days. Results are shown as the relative expression to GAPDH (mean ± SD), and significance was determined by Student's t-test. (* $p<0.05$ and ** $p<0.01$). Bar = 200 μm.

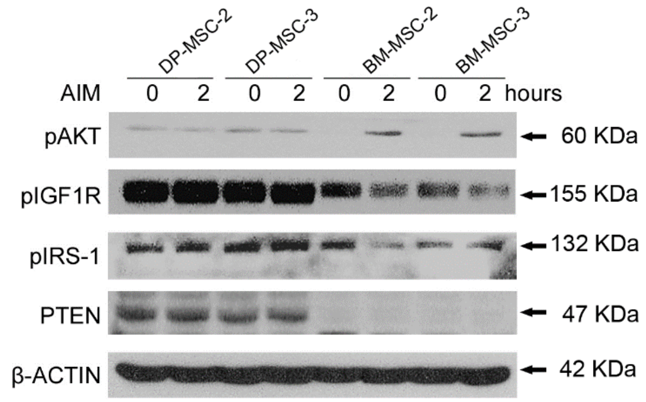

Supplementary Figure 3. DP-MSCs and BM-MSCs differ in insulin signaling. DP-MSCs and BM-MSCs were separately isolated from two other different individuals. Cells were analyzed by western blotting.

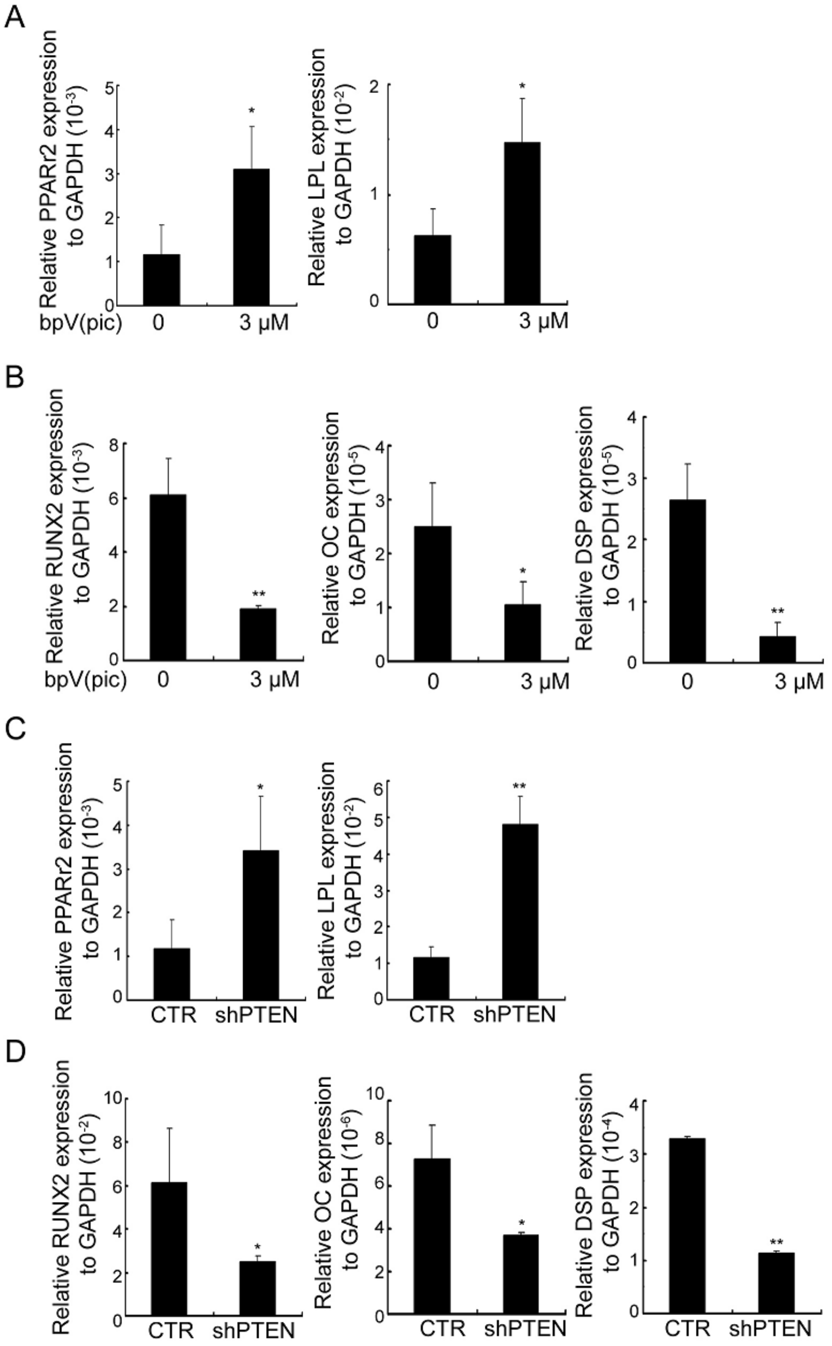

Supplementary Figure 4. Inhibition or knockdown of PTEN increases adipogenesis but decreases osteogenesis. (A-B) DP-MSCs were pretreated without or with a inhibitor of PTEN activity, bpV(pic), for 2 h, followed by induction in (A) adipogenic or (B) osteogenic induction medium for 7 days. (C-D) DP-MSCs without (CTR) or with PTEN knockdown (shPTEN) were induced in (C) adipogenic or (D) osteogenic induction medium. (A-D) Quantitative RT-PCR analysis of mRNA levels. Results are shown as the relative expression to GAPDH (mean ± SD), and significance was determined by Student's t-test. *, $p < 0.05$; **, $p < 0.01$ versus bpV(pic) = 0 or CTR *determined* by Student's t-test.

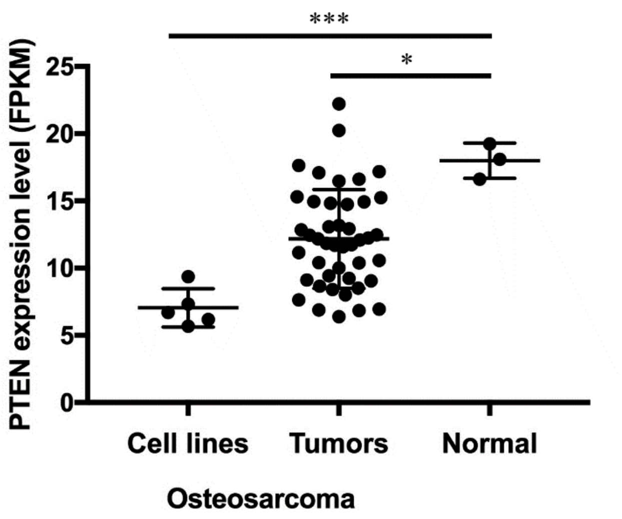

Supplementary Figure 5. PTEN levels in human normal bone and osteosarcoma tumors. The horizontal lines represent mean ± standard deviation for each group.

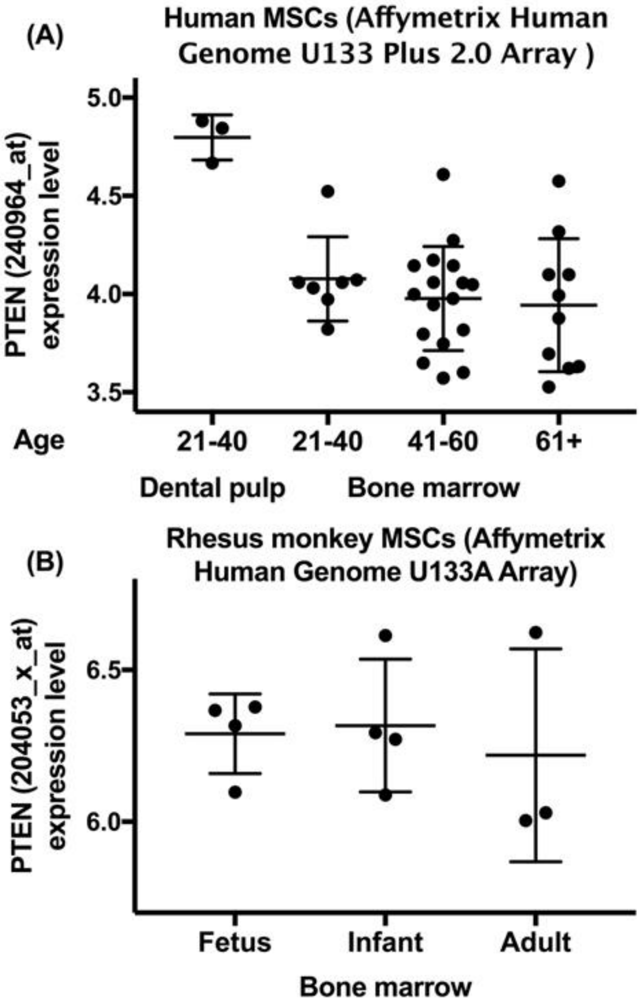

Supplementary Figure 6. PTEN levels in different MSC cell lines. PTEN levels at different ages from (A) human DP-MSCs and BM-MSCs, and (B) rhesus monkey BM-MSCs. For the same age range (i.e. from 21 to 40 years-old), human PTEN levels in DP-MSCs are significantly higher than those in BM-MSCs (t-test, $t_8 = 5.355$, P-value = 0.0007). In human BM-MSCs, PTEN is not differentially expressed among different age groups (ANOVA, $F_{2, 31} = 0.494$, P-value = 0.615). For rhesus monkey BM-MSCs, PTEN expression levels among different developmental stages are similar (ANOVA, $F_{2, 8} = 0.154$, P-value = 0.860). The horizontal lines represent mean ± standard deviation for each group.

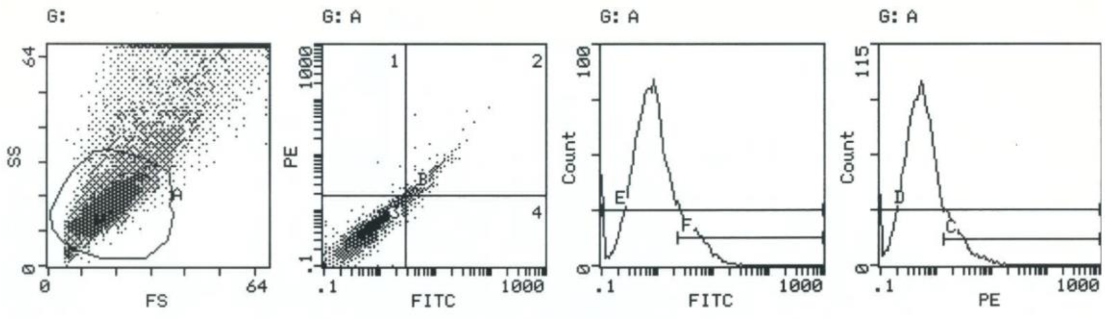

Supplementary Figure 7. Gating strategy for analyzing MSC flow cytometry data. After staining with antibodies or isotype antibodies, cells were resuspended in fixation buffer prior to flow cytometric analysis. The first step in gating is distinguishing populations of cells based on their forward (FS) and side scatter (SS) properties. To exclude debris and dead cells, particles with FS less than 52 were discarded. The circled area in the left panel represents the usual gating area. The events or cells within the gate were further analyzed for expression of markers. The percentages of cell positive for FITC or PE were counted. The histograms in the right panels were used to evaluate the relative expression of a marker (%) by subtracting the percentage of cells stained by the isotype antibodies (1 or 5%). The flow cytometry analysis was performed in triplicate.

Supplementary Table 1. Significantly enriched IPA diseases and bio functions

Diseases and Bio Functions	Gene
Differentiation of adipocytes (102 genes, p-value = 8.65 X 10^{-17})	ADIRF, ADM, ADRB2, ALDH6A1, ARL4A, ASXL2, BMP2, BNIP3, CAVIN1, CCNY, CEBPB, CEBPD, COPRS, CPXM1, CREB5, DACT1, DDIT3, EBF1, EGLN1, EGR2, ENPP1, EP300, FGF1, FGF2, FN1, FOXC2, FOXO1, GATA2, GDF6, GLIS3, GPC4, GRK5, HES1, ID2, ID4, IFI16, IL11, INSIG1, IRS1, JUP, KLF4, LAMB3, LRRC8C, MAP3K12, MEDAG, MMP1, MMP3, MSX2, MYC, NFE2L2, NIPBL, NOCT, NOTCH1, NR4A2, NR4A3, NUDT7, PEG10, PIAS1, PIK3R1, PPARA, PPARG, PRKG1, PTGFR, PTGS2, PTPRQ, RGS2, RNASEL, RORA, RUNX1T1, RUNX2, SAV1, SCD, SELENBP1, SEMA3A, SERPINF1, SFRP1, SIK3, SMAD7, SNAI1, SOCS1, SOD2, SOX11, STK4, TBL1X, TBX15, TCF7L2, TIMP3, TRIB2, TRIB3, TRPM4, UCHL3, VASP, VDR, WNT5A, XBP1, ZC3H12A, ZFP36, ZFP36L1, ZFP36L2, ZFPM2, ZNF385A, ZNF516
Differentiation of bone cells (152 genes, p-value = 5.08 X 10^{-20})	ACVR2A, ACVR2B, ADAM8, ADAR, ADRB2, ALPL, AREG, ASXL2, BGN, BMP2, BMP4, BMP8A, BMPER, C3, C3AR1, CAMK4, CAT, CBFB, CBL, CCR1, CD200, CD47, CD9, CEBPB, CEBPD, CSF1, CSF1R, CXCL8, CXXC5, CYR61, DKK1, EFNB1, EFNB2, EGFR, EIF2AK2, ENPP1, EPHB4, FAM20C, FAM213A, FBN2, FGF2, FGFR2, FHL2, FN1, FOS, FOXP1, FSTL3, GLI3, GLIS3, GPNMB, GPR68, GREM1, GTPBP4, HAND2, HDAC4, HGF, HIVEP3, HOXA10, HSPE1, HTRA1, IARS, ID2, ID3, ID4, IFI16, IFITM1, IFNGR1, IGFBP5, IL11, IL23A, IL6R, INHBA, INPP4B, IRAK3, IRS1, ITGA11, ITGA5, JUN, KITLG, KLF4, LGALS9, LPAR1, LRP4, MAP2K1, MAP2K3, MDK, MITF, MKX, MSX2, MYC, MYD88, NAMPT, NOCT, NOG, NOTCH1, NOV, PDGFRA, PDK4, PDLIM7, PIK3R1, PLCL2, POSTN, PPARG, PRKCD, PTCH1, PTEN, PTGER2, PTGS2, PTHLH, PTN, RACK1, RAPGEF3, RASSF2, RRAS2, RUNX2, SEMA3A, SEMA4D, SEMA7A, SERPINF1, SFRP1, SH3BP2, SIRPA, SMAD7, SNAI1, SOCS1, SOCS3, SOX11, STAT1, STAT6, STC1, SUFU, TCIRG1, TFRC, TGFB2, TGFBR3, TLR3, TMEM178A, TNC, TNFRSF11B, TNFSF10, TNFSF12, TOB2, TP53INP2, TPM4, TRPM4, TWIST2, TWSG1, UBASH3B, VCAN, WISP1, WNT5A, WNT7B, ZNF521
Bone marrow neoplasm (405 genes, p-value = 6.05 X 10^{-18})	AARS, ABCA1, ABCB1, ABCB4, ABCC1, ABCG2, ABHD12B, ACAN, ACE, ACSL4, ACTB, ADAMTS12, ADAMTS5, ADAMTS9, ADCY7, ADORA1, ADRA2C, ADRB2, AGAP6 (includes others), AKAP13, ALDH4A1, ANGPT1, ANGPTL2, ANK3, ANKFY1, ANKRD18B, ANKRD30B, ANKRD53, ANO4, APAF1, APCDD1, APOBEC3D, ARHGAP26, ARHGAP31, ARID4A, ARID4B, ATN1, ATP6V1A, ATP8A2, ATP8B1, ATRX, B4GALNT1, B4GALT2, BAALC, BAG1, BCL2L11, BCL2L13, BMP1, BMP2, C20orf96, C3, C5orf42, C8orf4, CADM1, CAND2, CARD14, CASP1, CBFB, CBL, CBLB, CD14, CD200, CD44, CD68, CD74, CDA, CDCP1, CDKN2B, CEBPB, CELSR3, CEP192, CHD2, CHL1, CHPF, CLDN7, CLIC3, CNTNAP3B, CPT1A, CREBBP, CSF1R, CSPG4, CTNND2, CYCS, CYP2R1, DAB2IP, DCBLD2, DCK,

	DDX58, DDX60, DDX60L, DENND3, DENND4C, DISP1, DMD, DNAH5, DNAJA1, DNMT3A, DOK1, DPP4, EBF3, EEF1A1, EGFLAM, EGFR, EIF2AK2, EIF3I, ENG, ENO2, EP300, EPB41L4B, EPHA3, EPHB1, EPHB2, EPHX1, EPOR, ERCC2, ERG, ETV5, EVC, F10, F3, FAT4, FGF2, FGFR2, FHOD3, FLG, FLNA, FLT1, FOXF1, FOXO1, FOXP1, GABBR2, GADD45A, GAREM1, GATA2, GBP2, GCC2, GDF5, GFAP, GHR, GLS, GOLGA8A/GOLGA8B, GPC4, GPRASP1, GREM1, GRIN3B, HAPLN3, HECW1, HES1, HLA-B, HLA-C, HMCN1, HOXA11, HOXA7, HOXA9, HOXB3, HSPA5, HSPD1, IARS, ID4, IFI44L, IGF2BP3, IL1R1, IL1RAP, IMPDH2, INSC, INTU, IRF1, IRS1, ITGA4, ITGB5, ITPR1, JAK2, JAK3, JUN, KAZN, KCNG1, KIAA1456, KITLG, KLF4, KMT2A, KMT2C, KMT2D, KRT18, KRT81, KSR1, L3MBTL1, LAMA1, LARS2, LDHB, LOXL3, LTBP4, LUM, MAMDC2, MAML3, MAP2K1, MASTL, MCM6, MLF1, MLLT3, MMP1, MMP12, MR1, MRPL37, MSI2, MSR1, MST1, MSX2, MTMR11, MXRA5, MYC, MYL9, MYO18A, MYO1E, MYO7B, NACAD, NAP1L3, NCKAP5, NCSTN, NFASC, NID1, NINL, NOG, NOTCH1, NPM1, NPR2, NR1H3, NSD3, NUMA1, NYNRIN, OBSCN, OPCML, OXER1, PADI1, PAMR1, PARP4, PCDHGB7, PDE1C, PDE3A, PDE4A, PDE4B, PDE4C, PDE4DIP, PDE7B, PDE8A, PDGFC, PDGFRA, PDGFRB, PDK4, PENK, PFKFB3, PGM1, PHACTR1, PHYHD1, PIAS1, PICALM, PIK3CD, PIK3R1, PKP3, PLCE1, PLCL2, PLOD2, PLXNA4, PLXNB1, PLXNC1, PML, POLE4, POR, PPP1R13L, PPP1R14B, PPP1R3B, PPP3CB, PPP3CC, PRKAA2, PRR5, PSEN2, PSMB10, PSME1, PSME2, PTEN, PTGS1, PTGS2, PTPRA, PTPRD, RACK1, RANBP17, RARA, RBM41, RDH10, REV3L, RHOA, RHOB, RIPK3, RPL13, RPL13A, RPL15, RPL3, RPL34, RPL35, RPL37, RPL4, RPL41, RPL5, RPL6, RPL7, RPLP0, RPLP1, RPS12, RPS13, RPS14, RPS15A, RPS16, RPS19, RPS20, RPS23, RPS24, RPS25, RPS27A, RPS3A, RPS4X, RPS6, RUNX1, RUNX1T1, RUNX2, RUNX3, RYR3, SALL2, SAMD8, SAMD9, SARS, SBDS, SCARB2, SCN9A, SEPT8, SERF2, SERINC2, SERPINE1, SESN2, SETBP1, SETD1B, SH3BP1, SIPA1, SIPA1L2, SKP2, SLC25A5, SLC7A7, SLC7A8, SLCO3A1, SMAD7, SMAD9, SMYD2, SMYD3, SORCS2, SOX4, SPEN, SRCAP, STAG2, STAT3, STAT5A, SV2A, SVEP1, SYCP2, SYNE2, SYNPO2, TAOK3, TAP2, TBC1D2, TCF12, TENM2, TET2, TGFBR1, THBS1, TLR1, TMCO4, TMEM132A, TMEM158, TMEM220, TMSB10/TMSB4X, TNC, TNFAIP6, TNFRSF10C, TNFSF10, TOP1, TPM2, TRERF1, TRIM6, TRIO, TSHZ3, TSPO, TUBE1, ULK4, UVSSA, VPS13D, VSTM4, WASH3P, WASHC2A/WASHC2C, WLS, XBP1, XPOT, ZC3H12C, ZC3H7B, ZFP36L2, ZFYVE26, ZNF107, ZNF217, ZZEF1

Supplementary Table 2. Donor information

Donor No.	Age (years)/ gender	Source of stem cells
DP1	5 / Female	lower anterior decious tooth (81)
DP2	5 / Male	lower anterior decious tooth (71)
DP3	6 / Male	lower anterior decious tooth (71)
BM1	27 / Female	Bone marrow
BM2	28 / Male	Bone marrow
BM3	41 / Male	Bone marrow

Supplementary Table 3. Primer sets for PCR and real-time PCR

Name	Primer sequence	Size	NCBI reference sequence
**PCR**			
GAPDH	F:GCACTCTTCCAGCCTTCCTTCC	515	NM_002046.3
	R:TCACCTTCACCGTTCCAGTTTTT		
PPARγ2	F:CCTATTGACCCAGAAAGCGATTC	595	NM_015869.4
	R:GCATTATGAGACATCCCCACTGC		
LPL	F:TGTAGATTCGCCCAGTTTCAGC	490	NM_000237.2
	R:AAGTCAGAGCCAAAAGAAGCAGC		
RUNX2	F:GTTTGTTCTCTGACCGCCTC	318	NM_004348.3
	R:CCAGTTCTGAAGCACCTGA		
OC	F:CGCAGCCACCGAGACACCAT	405	NM_199173.4
	R:GGGCAAGGGCAAGGGGAAGA		
AGGRECAN	F:TGAGGAGGGCTGGAACAAGTACC	350	NM_103227.3
	R:GGAGGTGGTAATTGCAGGGAACA		
COL2A1	F:CCAGGACCAAAGGGACAGAAAG	400	NM_033150.2
	R:TTCACCAGGTTCACCAGGATTG		
**real-time PCR**			
GAPDH	F:CTCTGCTCCTCCTGTTCGACA	112	NM_002046.3
	R:ACGACCAAATCCGTTGACTC		
PPARγ2	F:CTTCCATTACGGAGAGATCCAC	125	NM_015869.4
	R:AAGCGATTCCTTCACTGATACAC		
LPL	F:TACCCAGTGTTTGGGGTGTT	145	NM_000237.2
	R: GGATGTGCTATTTGGCCACT		
RUNX2	F:GGAGTGGACGAGGCAAGAGTTT	133	NM_004348.3
	R:AGCTTCTGTCTGTGCCTTCTGG		
OC	F:GACTGTGACGAGTTGGCTGA	119	NM_199173.4
	R:CTGGAGAGGAGCAGAACTGG		
DSP	F:GGG ATG TTG GCG ATG CA	70	NM_014208.3
	R:CCA GCT ACT TGA GGT CCA TCT TC		
