# Supplementary material for: Methylation and PTEN activation in dental pulp mesenchymal stem cells promotes osteogenesis and reduces oncogenesis
Source: Nat Commun. 2019 May 20;10:2226. doi: 10.1038/s41467-019-10197-x (PMC6527698; doi:10.1038/s41467-019-10197-x)
Supplement: Supplementary file 4 — Description of Additional Supplementary Files [file 41467_2019_10197_MOESM4_ESM.pdf]

Description of Additional Supplementary Files

File Name: Supplementary Data 1
Description: The differentially expressed gene list between BM-MSCs and DP-MSCs

File Name: Supplementary Data 2
Description: The sample list of mesenchymal stem cells (MSCs) from human and rhesus monkey
